# Long‐Acting Injectable Antipsychotic Use and Discontinuation Rates in Children and Adolescents With Schizophrenia Using Medicaid Claims Data

**DOI:** 10.1111/eip.70063

**Published:** 2025-06-17

**Authors:** Taylor M. Ward, Jianing Xu, Daniel B. Hall, Xianyan Chen, Sandra Benavides, Henry N. Young, Joshua Caballero

**Affiliations:** ^1^ College of Pharmacy University of Georgia Athens Georgia USA; ^2^ Department of Statistics Franklin College of Arts and Sciences, University of Georgia Athens Georgia USA; ^3^ KnowFully Medical Education Radnor Pennsylvania USA; ^4^ Department of Clinical and Administrative Pharmacy College of Pharmacy, University of Georgia Athens Georgia USA

**Keywords:** adherence, antipsychotics, long‐acting injectable, paediatrics, prescribing, racial disparity, schizophrenia

## Abstract

**Introduction:**

The primary objective was to analyse the prescribing and discontinuation rates of long‐acting injectable (LAI) antipsychotics among child and adolescent populations. The secondary objective was to assess if racial/ethnic differences existed between LAI antipsychotics and discontinuation rates.

**Methods:**

Children and adolescents (2–17 years old) with schizophrenia or related disorders who received LAI antipsychotics between January 1, 2017 and December 31, 2021 were identified using Merative MarketScan Multi‐State Medicaid Database. Descriptive statistics summarised the rates of LAI antipsychotic use. Kaplan–Meier survival curves were examined, and Cox regression analyses conducted to compare the hazard of discontinuation across LAI antipsychotics (*p* < 0.05).

**Results:**

A total of 1277 out of 67 502 patients were included in the final analysis. The average age was 15.4 ± 1.7 years (range 7–17 years). Approximately 59% were male, with the most common races identified being Black (48%) and White (38%). Prescribing of LAI second‐generation antipsychotics occurred in about 94% of the population. The most common LAI antipsychotics prescribed included paliperidone palmitate 1 month (40%) and aripiprazole formulations (48%). When controlling for age group, gender and plan type, the discontinuation rate for paliperidone and aripiprazole formulations did not differ. However, LAI paliperidone palmitate was associated with a 46% lower hazard of discontinuation for White compared to Black populations (HR = 0.54; *p* = 0.01).

**Conclusion:**

Despite the limited sample, this study explored the frequency of prescribing and discontinuation rates between LAI antipsychotics in children. Future studies may further explain the unique challenges (e.g., reasons for discontinuation) and economic impact LAI antipsychotics present.

## 1 Introduction

Over the last decade, mental health in child and adolescent patients has been increasing (Bitsko et al. 2022). Among these disorders, childhood‐onset schizophrenia, while rare may be the most debilitating (Bartlett 2014). Childhood‐onset schizophrenia typically manifest before the age of 13 with an estimated prevalence of approximately one in 40 000 children (Gochman et al. 2011; Kao and Liu 2010). Early onset schizophrenia is usually diagnosed prior to 18 years of age (Kao and Liu 2010). Overall, diagnosing schizophrenia in child and adolescent populations is difficult and may present with more negative symptoms and cognitive impairment compared to adult‐onset schizophrenia (Gochman et al. 2011; Hoff et al. 1996; Kravariti et al. 2003; Nicolson et al. 2000; Vourdas et al. 2003). Overall, patients experiencing early‐onset schizophrenia may have higher rates of premorbid abnormalities, poorer cognition and worse functional outcomes compared to patients developing schizophrenia at a later stage (Kao and Liu 2010). Regardless, child and adolescent patients may display both positive (e.g., aggression, hallucinations) and negative (e.g., impaired cognition, reduced emotional expression) symptoms. The primary medication treatment option for schizophrenia and related disorders is generally antipsychotics (Bartlett 2014). Data suggest paediatric patients respond better to SGA than FGA when used for schizophrenia spectrum disorders (Correll et al. 2021). This may be expected given that as mentioned earlier, early onset schizophrenia may have high rates of negative symptoms and cognitive impairment (Hoff et al. 1996; Kravariti et al. 2003; Nicolson et al. 2000; Vourdas et al. 2003). For example, in comparative paediatric studies, there was a large effect size favouring SGA over FGA (Correll et al. 2021). The side effect profile between FGA and SGA also differ. Motor complications (e.g., akathisia, pseudo‐parkinsonism) appear to be more common with FGA while weight gain, sedation, prolactin elevations and constipation may be seen more often with SGA (Sikich et al. 2008). Based on efficacy and side effect profiles, there appears to be a preference to using SGA in child and adolescent populations (Correll et al. 2021; Hayes and Kyriakopoulos 2018).

While data are available for oral antipsychotics in the treatment of schizophrenia in younger patients, there is a scarcity of knowledge regarding long‐acting injectable (LAI) antipsychotics. Typically, LAI antipsychotics are generally used in patients with schizophrenia or other mental health disorders who have difficulty with adherence (Hojlund and Correll 2023; Ostuzzi et al. 2022; Pope and Zaraa 2016). LAI antipsychotics are designed to improve patient‐centred care and encourage medication adherence and reduce relapse (Ostuzzi et al. 2022). LAI antipsychotics work by slowly releasing medication into the body via site of injection over weeks to months. This allows for more consistent blood concentrations that are less frequently affected by missed dosing or pill burden. The use of LAI antipsychotics in adults may decrease rehospitalisations, increase adherence and reduce medical costs (Huang et al. 2021; Kishimoto et al. 2021; Lin et al. 2021). Different formulations (e.g., aripiprazole lauroxil, aripiprazole monohydrate) and dosing frequencies are available for several of these agents. Current United States Food and Drug Administration (FDA) approved LAI antipsychotics include both LAI FGA (e.g., haloperidol decanoate, fluphenazine decanoate) and LAI SGA (e.g., olanzapine pamoate, risperidone microspheres/subcutaneous, aripiprazole monohydrate/lauroxil and paliperidone palmitate) (VandenBerg 2022).

Most clinical trials on the effectiveness and efficacy of LAI antipsychotics are studied in adult patients, but limited studies are available for child and adolescent populations. It appears SGA are the most common class of antipsychotics prescribed by physicians for child and adolescent psychiatric disorders (Llorca et al. 2013; Seida et al. 2012). Preliminary data on prescribing rates from approximately 10 years ago show some LAI antipsychotics used in child and adolescent patients predominantly include paliperidone palmitate, risperidone microspheres and aripiprazole (Modesitt et al. 2018). However, it is limited to the state of Indiana; thus, it is unknown if these patterns are consistent with the rest of the United States. Additionally, discontinuation data between agents are lacking. While there are no FDA‐approved LAI antipsychotics for child and adolescent patients, they are used off label to treat varying psychotic disorders in child and adolescent patients. There are some data describing the use (e.g., efficacy, adherence) of LAI antipsychotics in child and adolescent patients (Benarous et al. 2022; Chowdhury et al. 2024; Moon et al. 2023). However, they are limited by small sample sizes (e.g., less than 50 patients), limited comparison between LAI agents, and possible bias of including child and adolescent patients with schizophrenia and other psychiatric complications (e.g., bipolar) which may confound the results. Additionally, while use and discontinuation rates between LAI antipsychotics and race/ethnicity have been described with adults (Caballero et al. 2023; Pesa et al. 2023), no data are available in patients younger than 18 years of age. As a result, there is a need to address the lack of data centred on comparing the discontinuation rates between LAI antipsychotics in child and adolescent patients. Therefore, the primary objective of the study was to identify the prescribing rates of LAI antipsychotics among child and adolescent populations and evaluate discontinuation rates between agents. Secondary objectives were to determine if racial/ethnic differences existed between LAI antipsychotics and discontinuation rates.

## 2 Methods

### 2.1 Study Design and Data Sources

Data were evaluated between January 1, 2017–December 31, 2021 using Merative MarketScan Multi‐State Medicaid Databases (referred to hereafter as the MarketScan Medicaid Database). Panel records of prescription drug claims along with inpatient and outpatient services for millions of patients can be found in the MarketScan Medicaid Database. The MarketScan Medicaid Database reflects the healthcare service use of individuals covered by Medicaid programmes in numerous geographically dispersed states. Medicaid is a joint federal and state programme that provides health coverage to eligible low‐income individuals and families. Eligibility criteria vary by state and are based on factors such as income, age, disability status and family composition. The database contains the pooled healthcare experience of Medicaid enrolees, covered under fee‐for‐service and managed care plans. It includes records of inpatient services, inpatient admissions, outpatient services and prescription drug claims, as well as information on long‐term care. The data contain information surrounding treatment patterns among varying patient populations. Demographic characteristics in the MarketScan Medicaid Database are determined through patient self‐identification. The MarketScan Medicaid Database contains between 11.5 and 14.2 million people per year. The number of younger patients (2–17 years of age) accounts for approximately 40% of the population with approximately 5 million people per year. Among this younger population, children (2–11 years) account for approximately 26% and adolescents (12–17 years) account for approximately 14% of the population within the years 2017–2021. International Classification of Diseases, Tenth Revision, Clinical Modification (ICD‐10‐CM) diagnosis codes and health plan information were collected via medical claims provided by providers and facilities. Subsequently, National Drug Code (NDC) numbers for dispensed LAI antipsychotic medications were collected from pharmacy claims. IRB exempt status was attained through the University.

### 2.2 Study Population and Cohort Assignment

Patients between the ages of 0–17 with a pharmacy claim for a LAI antipsychotic were identified in the MarketScan Medicaid Database. NDC numbers (https://ndclist.com/) were used to identify LAI antipsychotics and doses using brand/generic names and, when necessary, cross‐referenced with the package insert of the medication through the United States FDA drug database (www.accessdata.fda.gov). Patients were required to have ≥ 1 inpatient or ≥ 2 outpatient claims with an ICD‐10‐CM diagnosis code for schizophrenia (F20.x), schizotypal (F21) or schizoaffective (F25.x). The index date was established as the date of their first LAI antipsychotic prescription, which had to occur subsequent to the initial diagnosis. A refill date was termed as the fill date plus the coverage period. The *fill date* is the date when the prescription medication is dispensed by the pharmacy and represents the starting point for the medication. The *refill date* refers to the specific date a prescription medication is refilled. The *coverage period* refers to the timeframe over which the prescription medication supplied is expected to last. For example, 30‐day supply should cover a period of 30 days following the fill date. A *continuous medication gap* is the defined period during which a patient has no medication supply available based on the coverage period of a prior fill. The refill date varied based on the medication and formulation type (e.g., 2 weeks vs. 4 weeks for different risperidone formulations). As observed in prior studies with similar methodology using a conservative approach, discontinuation rates were determined as a continuous medication gap of 60 days or greater after the last day of LAI antipsychotic coverage (Lauriello et al. 2021; Marcus et al. 2015).

In summary, Medicaid enrolment status was assessed for each patient during the month coinciding with the last day of LAI antipsychotic coverage, specifically for patients who experienced a continuous medication gap of 60 days or more. Similar to other studies, enrolment status was determined using monthly enrolment data from the Marketscan Merative Database (Caballero et al. 2023; Chiang et al. 2024). Patients were considered to have “inadequate enrolment” if they were not actively enrolled in Medicaid during the relevant month, as indicated by a lack of recorded Medicaid insurance coverage or a lapse in consecutive monthly enrolment. Such patients were not classified as having a discontinuation of medication; instead, they were censored from the analysis due to the inability to confirm whether the observed medication gap was due to true discontinuation or a lapse in insurance coverage.

### 2.3 Study Variables

The main variable of interest was the duration from the index date to discontinuation date, which was defined as the number of days each prescription fill would last based on its formulation. Discontinuation rates for antipsychotics were measured for LAI FGA (i.e., haloperidol decanoate and fluphenazine decanoate) and LAI SGA (i.e., aripiprazole monohydrate, aripiprazole lauroxil, risperidone microspheres, risperidone subcutaneous, olanzapine pamoate and paliperidone palmitate 1 month formulation). The Medicaid health plans for this observed population included: Comprehensive, health maintenance organisation (HMO), or both. Patient demographics (i.e., age, gender and race/ethnicity) and type of health plan were collected at the initial index date. Race/ethnicity was classified as Black, Hispanic, White or other, and gender was defined as female or male. Based on Food and Drug Administration legislative guidance (Pediatric Drug Development 2023), age was separated into two groups: children (2–11 years) and adolescents (12–17 years).

### 2.4 Statistical Analysis

Descriptive statistics were used to determine demographic characteristics across our cohorts. To evaluate the association between race/ethnicity and time to discontinuation of LAI antipsychotics, Cox proportional hazards (CoxPH) models were completed adjusting for potential confounders, including age at first diagnosis, gender and plan type. Kaplan–Meier survival curves were examined, and Cox regression analyses were conducted to compare the hazard of discontinuation of LAI antipsychotics (*p* < 0.05).

## 3 Results

A total of 1274 out of 67 502 patients met the inclusion criteria and were included in the final analysis. There were approximately 97% adolescents (*n* = 1230) and 3% children (*n* = 44) with a mean age of 15.35 (SD; ±1.72) years and an age range of 7–17 years of age. Approximately 59% were male (*n* = 745) with approximately 48% Black (*n* = 606), 38% White (*n* = 489), 3% Hispanic (*n* = 38), 3% other (31) and 9% missing (*n* = 110). Prescribing of LAI SGA occurred in about 94% of the population. The most common LAI SGA antipsychotics prescribed included paliperidone palmitate 1 month (40%), aripiprazole monohydrate (38%) and aripiprazole lauroxil (10%). Of note, there was no use of LAI olanzapine pamoate in this population (see Table 1). Prescribing rates between Black and White populations and these agents did not differ.

**TABLE 1:** Use of long‐acting injectable antipsychotics in child and adolescent patients.

Antipsychotic	Sample size (%)
Paliperidone palmitate 1 month	508 (39.9)
Aripiprazole monohydrate	483 (37.9)
Aripiprazole lauroxil	129 (10.1)
Haloperidol decanoate	67 (5.3)
Risperidone microspheres	65 (5.1)
Fluphenazine decanoate	12 (0.9)
Risperidone subcutaneous	10 (0.8)
Olanzapine pamoate	0 (0)

**FIGURE 1:**
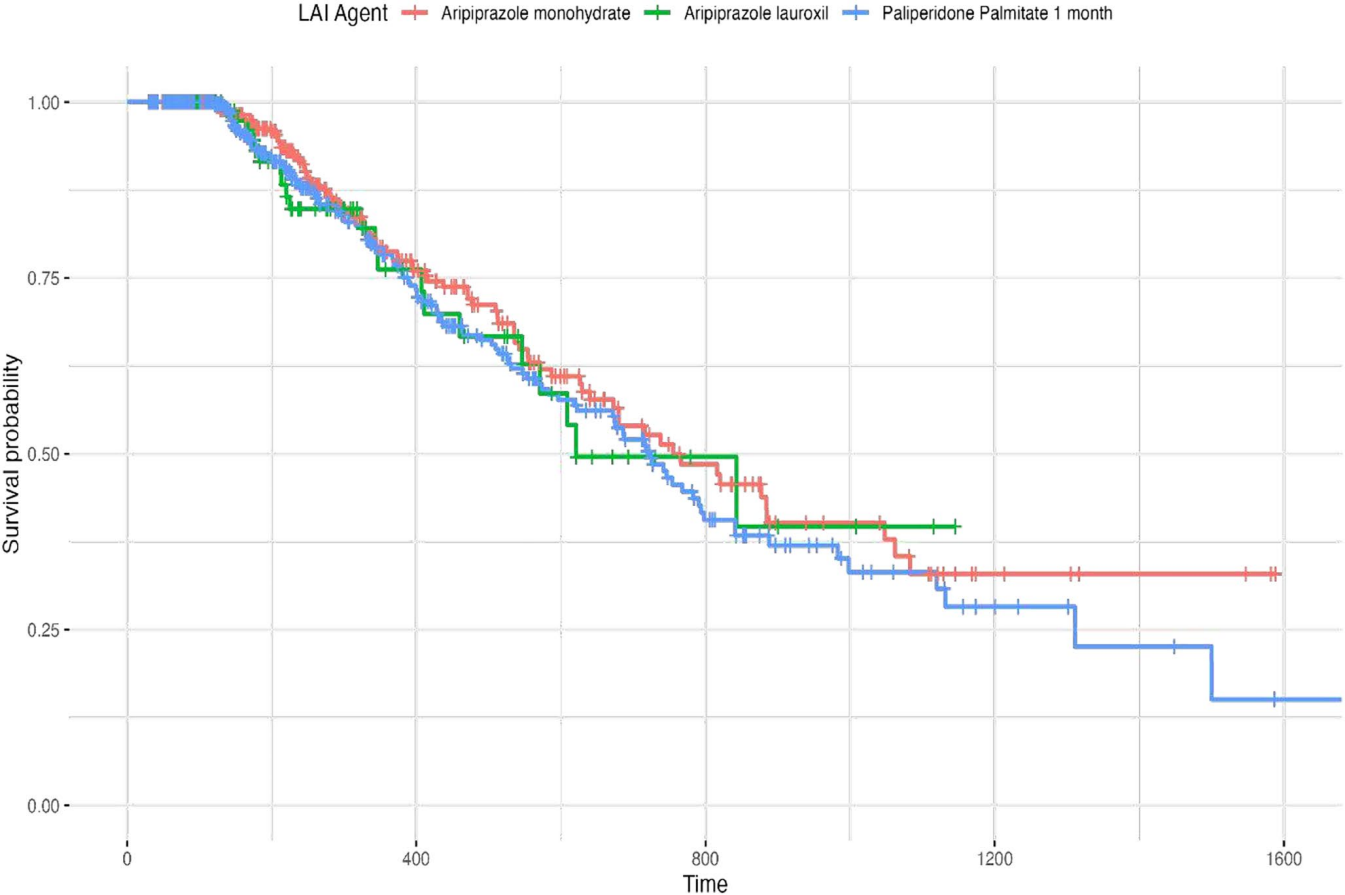
Discontinuation rates for LAI antipsychotics in child and adolescent patients. LAI, long‐acting injectable.

**FIGURE 2:**
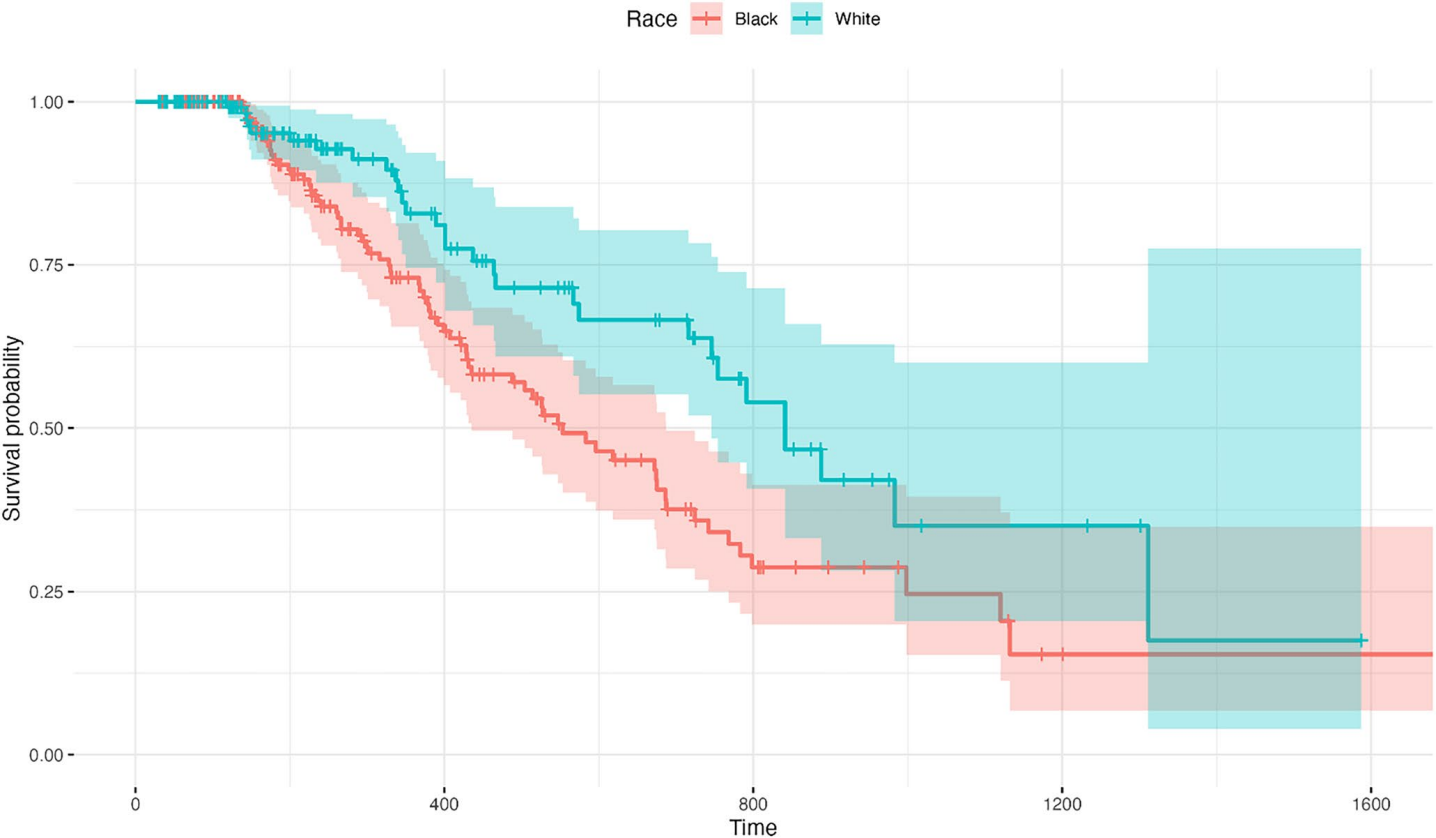
LAI paliperidone palmitate 1 month discontinuation rates for Black compared to White populations*. *Statistically significant difference (HR = 0.54; *p* = 0.01). LAI, long‐acting injectable.

When controlling for age group, gender and health plan type, the discontinuation rate for LAI paliperidone palmitate 1 month and LAI aripiprazole formulations (i.e., aripiprazole monohydrate, aripiprazole lauroxil) did not differ. A sub‐analysis focusing solely on the adolescent population also showed no differences in discontinuation rates. Based on the populations sizes (White and Black populations having largest sample sizes), discontinuation rates of LAI antipsychotics between Black and White populations were further explored for paliperidone palmitate 1 month, aripiprazole monohydrate and aripiprazole lauroxil. While controlling for age group, gender and plan type, the data showed no difference in discontinuation rates between Black and White populations for paliperidone palmitate 1 month, aripiprazole monohydrate and aripiprazole lauroxil (see Figure 1). Additionally, there were no differences between Black and White populations and aripiprazole formulations (i.e., aripiprazole monohydrate and aripiprazole lauroxil). However, paliperidone palmitate 1 month showed an estimated hazard ratio (HR) of 0.54 (95%, CI: 0.35–0.86, *p* = 0.01). Specifically, White individuals appear to exhibit approximately a 46% lower risk of discontinuing LAI paliperidone palmitate 1 month compared to Black individuals (see Figure 2). Analysis of other racial/ethnic populations were not completed due to lack of sufficient sample sizes to support inferential analysis.

**FIGURE 3:**
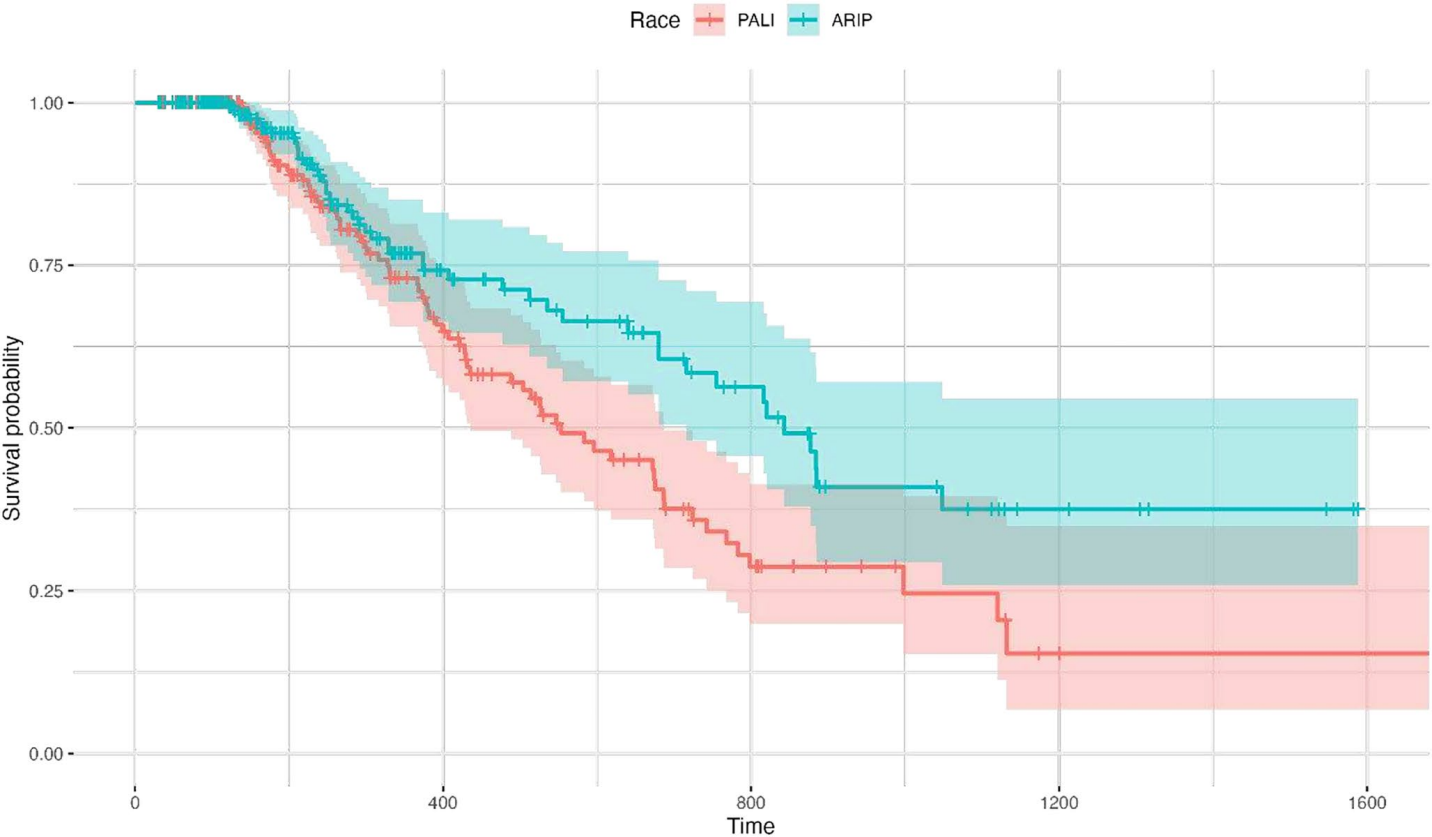
LAI paliperidone palmitate 1 month and aripiprazole formulations (i.e., aripiprazole monohydrate and aripiprazole lauroxil) discontinuation rates among Black individuals*. *Statistically significant difference (HR = 0.57; *p* = 0.004). ARIP, aripiprazole formulations; LAI, long‐acting injectable; PALI, paliperidone palmitate 1 month.

While controlling for age‐group, gender and plan type, further analysis showed discontinuation rates did not differ between paliperidone palmitate 1 month and aripiprazole formulations (i.e., aripiprazole monohydrate, aripiprazole lauroxil) among White individuals. However, the risk of discontinuation of LAI among Black individuals were significantly lower for aripiprazole formulations (i.e., aripiprazole monohydrate, aripiprazole lauroxil) compared to paliperidone palmitate 1 month (HR = 0.57; *p* = 0.004) (see Figure 3).

## 4 Discussion

Based on the data collected, LAI paliperidone palmitate 1 month, aripiprazole monohydrate and aripiprazole lauroxil were the most commonly prescribed agents in child and adolescent patients with Medicaid. This aligns with SGA oral antipsychotics currently approved in child and adolescent patients that are available in LAI formulations (Benarous et al. 2022; VandenBerg 2022). Some advantages to LAI antipsychotics can include a reduction in pill burden, increased adherence due to more clinical awareness of dosage administration, and greater quality of life (Correll et al. 2016; Pietrini et al. 2019). Other benefits may include but are not limited to increased safety and efficacy monitoring due to more frequent face‐to‐face interactions, and longer periods at steady state between doses compared to taking daily oral medications (Spanarello and La Ferla 2014). Some disadvantages may include cost burden due to higher cost per dose, increased visits to the prescriber's office and the time burden for caregivers who must take underage patients to their follow‐up appointments (Correll et al. 2016). Despite cost being a potential burden, this study narrowed its focus on patients who had a state Medicaid programme at the time of dose administration.

Among Medicaid claims, LAI SGA were more commonly prescribed with paliperidone palmitate 1 month and aripiprazole formulations accounting for approximately 88% of the total use. Overall, there were no differences in prescribing rates between races/ethnicities and agents used. The results suggest no variances in discontinuation rates among White patients. However, differences were noted for LAI paliperidone palmitate 1 month in which Black patients discontinued at a higher rate than White patients in a child and adolescent population. At this time, it is unknown why child and adolescent Black patients discontinue LAI paliperidone palmitate 1 month at a greater rate than child and adolescent White patients. However, based on previous data there could be some plausible hypotheses to explain the differences noted in this study.

First, extra‐pyramidal symptoms (EPS) may be reasons for the differences in discontinuation rates between Black and White populations taking LAI paliperidone palmitate 1 month and LAI aripiprazole formulations. This hypothesis is based on a few factors. For example, adult studies suggest Black patients may be more susceptible to developing EPS than White patients (Cook et al. 2015). Among the SGA, a meta‐analysis of adult studies showed oral paliperidone may be more likely to cause EPS compared to aripiprazole (Leucht et al. 2013). Previous studies in adult populations show that SGA LAIs have a similar side effect profile compared to oral SGA (Wang et al. 2024). Overall, among children and adolescents, SGA are prescribed more often than FGA and may be partly related to a preference for a lower risk of developing EPS (Altuwairqi 2024). More specifically, child and adolescent patients may be more sensitive to EPS, especially pseudoparkinsonism (Correll 2008; Correll et al. 2006). Paliperidone, while considered an SGA, has higher dopamine occupancy compared to aripiprazole and as a result, a greater risk for pseudoparkinsonim compared to aripiprazole (Kikuchi et al. 2021; Kozielska et al. 2012; Marino and Caballero 2008). Data in adults suggest a lower non‐significant risk for EPS with LAI aripiprazole compared to other LAI antipsychotics, including LAI paliperidone palmitate (Kannarkat et al. 2022). Given the literature provided, it may be possible race and age may play a role in Black children having a higher risk of discontinuing LAI paliperidone compared to aripiprazole. However, there appears to be a lack of direct prospective comparative studies between LAI paliperidone palmitate and any aripiprazole formulations in child and adolescent patients. Such studies may be needed to address any differences in efficacy, adverse events and reasons for discontinuing.

Metabolic or endocrine complications (e.g., weight gain, prolactin elevations) may be another explanation for showing differences in discontinuation rates between LAI paliperidone palmitate 1 month and LAI aripiprazole formulations. Studies appear to support that paliperidone is more likely to cause metabolic or endocrine complications than aripiprazole (Tasaki et al. 2021; Wu et al. 2022). In adult populations, LAI paliperidone palmitate has been compared to LAI aripiprazole (Cuomo et al. 2018; Schneider‐Thoma et al. 2022). In these studies, LAI paliperidone palmitate appeared more likely to be associated with hyperprolactinemia compared to aripiprazole. Additionally, LAI aripiprazole formulations were associated with little to no changes in body weight when compared to placebo (Cuomo et al. 2018; Schneider‐Thoma et al. 2022). Among child and adolescent populations, it appears that oral formulations of aripiprazole may offer less weight gain and prolactin elevations compared to paliperidone (Dayabandara et al. 2017; Krause et al. 2018; Pagsberg et al. 2017). Additionally, data suggest Black individuals may be more susceptible to weight gain and other metabolic parameters among SGA antipsychotics (Krakowski et al. 2009). Therefore, it may also be possible that Black child and adolescent patients may be more susceptible to metabolic side effects, which may lead to discontinuation differences favouring LAI aripiprazole formulations over LAI paliperidone palmitate. However, this also remains a hypothesis requiring further investigation between LAI antipsychotics side effects in child and adolescent populations and race/ethnic comparisons.

Side effects may not be the only reason for discontinuing LAI antipsychotics. Overall, LAI antipsychotics may bring on a large burden to the caregiver since they may be responsible for securing follow‐up appointments and providing transportation to a child and adolescent patient for their scheduled injection. Travel distance and operating hours of the LAI clinic or physician's office can increase this burden due to other responsibilities of the caregiver. Other factors that may affect discontinuation include change in provider, lack of knowledge regarding illness and side effects, or aversion to healthy lifestyle modifications (Aouira et al. 2023; Deng et al. 2022; El Abdellati et al. 2020). While some of these challenges appear to affect minority groups at a higher rate (Levy 2010), issues with side effects may be more likely to explain the differences in our results. For example, one study evaluating metabolic complications found child and adolescent patients have fears of needles (necessary to monitor metabolic blood parameters), and disliking eating health foods and exercising (Aouira et al. 2023). Therefore, it may be plausible that child and adolescent patients experiencing metabolic complications that may be more likely with paliperidone and among Black child and adolescent patients, may be discontinuing treatment at a higher rate.

### 4.1 Limitations

This study may have several limitations. First, the reasons for discontinuation between LAI antipsychotics mainly between paliperidone palmitate 1 month and aripiprazole formulations in this child and adolescent population cannot be determined. Also, there could be reporting biases and coding errors of omission and commission. While the study controlled for some variables (e.g., plan type, age and gender), other factors may have contributed to the differences found in the results. However, the study elucidated the use of these agents in caring for child and adolescent patients with schizophrenia or related disorders. Sample size could also be a limitation; however, there are a lack of published data on the use and discontinuation rates of LAI antipsychotics in child and adolescent populations and discussion among race and ethnicities. Furthermore, only paliperidone palmitate 1 month formulations was evaluated and further evaluation of longer acting formulations (e.g., 3, 6 month) may merit further investigation.

Finally, at this time MarketScan Medicaid Database does not provide state‐level identifiers, which may affect the ability to evaluate geographic representation and assess regional variations. Therefore, without state‐specific data, it is difficult to determine how differences in provider availability, insurance plan regulations, healthcare policies and regional or demographic factors between states may impact the findings of this study. As a result, this limitation may affect generalizability, since some states may be over‐ or underrepresented in the data, thereby possibly introducing a regional bias that may be difficult to quantify. Additionally, differences in Medicaid expansion, reimbursement plans and access to specialty care may affect variations in prescribing patterns that cannot be elucidated in our analysis. Despite this limitation, MarketScan Medicaid Database is a commonly used source of real‐world healthcare data, which provides comprehensive claims information from various populations across employer‐sponsored and Medicaid plans Prior recent studies using MarketScan Medicaid Database have been published by providing nationally relevant insights without state‐level stratification including studies assessing antipsychotic utilisation (Pesa et al. 2023; Chiang et al. 2024; Caballero et al. 2023). The database's national coverage across several payers and demographic groups provides an overall applicability of our findings. Therefore, the results of this study should encourage future research using state Medicaid databases to further identify state (or regional) specific effects and refine generalizability.

## 5 Conclusion

This study explored the prescribing rates and discontinuation rates between LAI antipsychotics in patients younger than 18 years of age. Overall, child and adolescent patients received a LAI SGA more often than LAI FGA, including paliperidone palmitate 1 month and aripiprazole formulations. Among the most commonly used agents (i.e., paliperidone palmitate 1 month and aripiprazole formulations), there did not appear to be overall differences in discontinuation rates. However, the differences found between race/ethnic child and adolescent populations suggest LAI aripiprazole formulations may be more acceptable over LAI paliperidone palmitate 1 month in child and adolescent Black patients. Regardless, these results merit further study that may explain the unique challenges (e.g., reasons for discontinuation) with LAI antipsychotic treatment in child and adolescent patients with schizophrenia and related disorders.

## Conflicts of Interest

The authors declare no conflicts of interest.

## Data Availability

The data that support the findings of this study are available from Merative MarketScan. Restrictions apply to the availability of these data, which were used under license for this study. Data are available from the author(s) with the permission of Merative MarketScan.
